# Hyperbilirubinemia and Hyponatremia as Predictors of Complicated Appendicitis

**DOI:** 10.3390/medsci10030036

**Published:** 2022-07-04

**Authors:** Abdullah Shuaib, Nour Alhamdan, Husain Arian, Mohamed Alaa Sallam, Ali Shuaib

**Affiliations:** 1 Department of General Surgery, Jahra Hospital, Al-Jahra 40206, Kuwait; abdullah.shuaib.77@gmail.com (A.S.); arianh@tcd.ie (H.A.); sm_alaa@hotmail.com (M.A.S.); 2 Department of Medicine, Jaber Al-Ahmad Hospital, Ministries Area 91710, Kuwait; nourgalhamdan@gmail.com; 3 Biomedical Engineering Unit, Department of Physiology, Faculty of Medicine, Kuwait University, Safat 13110, Kuwait

**Keywords:** complicated appendicitis, hyperbilirubinemia, hyponatremia, predictor

## Abstract

Several studies have reported elevated serum bilirubin or reduced serum sodium levels in patients with complicated appendicitis (CA). This study examined the efficacy of hyperbilirubinemia, hyponatremia, and both combined in the preoperative diagnosis of CA. Patients who underwent surgery for acute appendicitis were included in this retrospective review. In total, 247 patients were included in the final analysis. Of these, 36 (14.2%) had early appendicitis, 177 (72.0%) had acute suppurative appendicitis, 32 (13.0%) had necrotizing/gangrenous acute appendicitis, and 2 (0.8%) had other types of appendicitis. The mean total bilirubin (TBIL) level was significantly higher in patients with CA than in those with uncomplicated appendicitis. Conversely, the mean serum sodium level was significantly lower in patients with CA than in those with uncomplicated appendicitis. The levels of TBIL (odds ratio: 1.098, 95% CI: 1.052–1.147) and serum sodium (odds ratio: 0.743, 95% CI: 0.646–0.855) were associated with CA. Hyponatremia combined with hyperbilirubinemia yielded significant discriminatory value for the diagnosis of CA. TBIL and serum sodium levels can be considered as adjuvant parameters in the diagnosis of perforated/necrotizing appendicitis. Although hyperbilirubinemia and hyponatremia together were better able to determine the risk of CA than either marker alone, other markers are required to definitively predict CA. Furthermore, large-scale studies are needed to confirm these findings.

## 1. Introduction

Appendicitis is one of the most common reasons for emergency surgery [1]. The need for surgery depends on the diagnosis of the correct stage of acute appendicitis. Depending on the stage, appendicitis can either be self-limiting or warrant urgent surgical intervention [2,3]. Striking an appropriate balance between an unnecessary and a necessary appendectomy plays a deciding role in the overall clinical outcome of this procedure. In the case of an uncertain diagnosis, the general strategy is to observe the patient and to delay an appendectomy until a more definitive diagnosis is reached. However, given that its clinical presentation is usually inclusive and its preoperative diagnosis is rarely definitive, the successful management of acute appendicitis is complex [4,5,6].

Several laboratory tests, scoring methods, radiological assessments, and laparoscopic approaches have been studied with the aim of identifying potential markers of complicated appendicitis (CA) [1,5,7,8,9]. However, none of these methods can be used as standalone diagnostic tools, as shown by one study, which reported that more than 20% of appendicitis diagnoses were missed [10]. Computed tomography (CT) is the most useful tool for the diagnosis of acute appendicitis [11]. In a recent study, the pooled sensitivity of CT for the differentiation of complicated appendicitis from uncomplicated appendicitis was reported to be 92%; however, the pooled specificity was only 43% [12]. All these factors suggest that there is a need for further research on biomarkers and other techniques that could potentially be used for the reliable, efficient preoperative diagnosis of complicated appendicitis.

An imbalance between bilirubin output and hepatic excretion is thought to cause elevated serum albumin levels. However, only a few studies have examined preoperative hyperbilirubinemia in patients with complicated appendicitis [11,13,14]. Moreover, a broad range of sensitivity and specificity values have been observed in studies that used evaluated hyperbilirubinemia as a predictor of acute appendicitis [11,13,14]. These discrepancies may stem from the fact that an increase in bilirubin is observed not only in acute appendicitis but also in other inflammatory conditions of the abdomen. Differences in sample size and serum level cut-offs may also have contributed to differences in the reported results [15]. In a few studies, hyponatremia was also identified as an independent risk factor for acute appendicitis [16,17,18]. However, the preoperative diagnostic efficacy of hyponatremia in complicated appendicitis has not yet been fully established [19,20].

The present study examined the diagnostic efficacy of hyperbilirubinemia, hyponatremia, and both combined in the prediction of complicated appendicitis. The characteristics of patients with various stages of acute appendicitis were also investigated.

## 2. Materials and Methods

We performed a retrospective single-center study on patients who had undergone operations for acute appendicitis in the general surgery department at Jahra Hospital between January 2018 and January 2020. Data were extracted from these patients’ medical records, which were available in a computer-based hospital information system.

The inclusion criteria were as follows: admitted for acute appendicitis, underwent surgery, and postoperatively confirmed histopathology of acute appendicitis. The exclusion criteria were as follows: incomplete documentation in the medical record, no histopathological report, liver disease, liver transplantation, chronic alcoholism, congenital biliary disease, pregnancy, appendicular neoplasm, hemolytic disease, or known hepatocellular carcinoma.

The retrieved data included each patient’s sex, age, serum sodium level, total bilirubin (TBIL) level, histopathology, and type of surgery. Based on the histological findings, the stage of appendicitis was defined as early acute, acute suppurative, acute necrotizing gangrenous with perforation, or another type of appendicitis. Complicated appendicitis, characterized as intraoperative findings of acute necrotizing gangrenous appendicitis with perforation, was the primary outcome of interest. Hyperbilirubinemia was defined as a TBIL level of >20 µmol/L, and hyponatremia was defined as a serum sodium level of <135.0 mEq/L.

Ethical approval for the study was obtained from the Ministry of Health Research ethical committee, Kuwait (MoH/REC/1832-107), and the study was performed in accordance with the Declaration of Helsinki.

The statistical analysis was carried out using SPSS for Windows, version 20.0 (IBM Corp., Albany, NY, USA). Continuous variables are expressed as the means ± standard deviations, and categorical variables are expressed as absolute numbers and percentages. Prior to the statistical analysis, the data were tested for normality. An unpaired *t*-test and a Mann–Whitney U test were used to compare normally and non-normally distributed continuous variables. A chi-square test or Fisher’s exact test was used to evaluate each categorical variable. Univariate and multivariate logistic regression analyses were performed to evaluate the associations of sex, TBIL level, and serum sodium level with complicated appendicitis [21]. A receiver operating characteristic (ROC) curve analysis was used to measure the area under the curve (AUC) and the specificity and sensitivity of each marker and, thus, determine their diagnostic values. A *p*-value of <0.05 was considered to be statistically significant in all statistical analyses.

## 3. Results

Data were obtained from 289 patients who had undergone an appendectomy for acute appendicitis. Forty-two patients were excluded due to incomplete laboratory results or missing histopathological reports. Thus, the final study included 247 patients. The ratio of males to females was 1.71:1, and the mean (±SD) age of the patients was 24.6 ± 12.4 years.

The mean ± SD TBIL and sodium levels were 17.36 ± 7.87 µmol/L and 134.63 ± 2.82 mEq/L, respectively. The histopathological diagnosis of appendix specimens revealed that 36 (14.6%) patients had early appendicitis, 177 (71.7%) patients had acute suppurative appendicitis, 32 (13.0%) patients had complicated appendicitis, and 2 (0.8%) patients had other types of appendicitis (appendices with Enterobius vermicularis).

In total, 74 (30%) patients had hyperbilirubinemia, 119 (48.2%) patients had hyponatremia, and 48 (19.4%) patients had both hyperbilirubinemia and hyponatremia. Laparoscopic appendectomy was performed in 230 (93.1%) cases, and only 17 (6.9%) patients underwent open appendectomy. The incidences of complicated appendicitis and type of surgery were similar between male and female patients (all *p* > 0.05). The TBIL levels of the female patients were substantially lower than those of the male patients (mean ± SD: males vs. females, 18.3 ± 7.9 vs. 15.7 ± 7.6 µmol/L, *p* = 0.013). However, there was no sex difference in serum sodium levels (mean SD: males vs. females, 134.7 ± 2.9 vs. 134.6 ± 2.7 mEq/L, *p* = 0.872). In addition, the proportion of patients with hyperbilirubinemia and hyponatremia did not differ significantly between the male and female patients.

Patients with different stages of appendicitis had substantially different TBIL and serum sodium levels (*p* < 0.001). Specifically, TBIL levels showed an increasing trend, whereas serum sodium levels showed a decreasing trend as the severity of appendicitis increased from early to complicated appendicitis (Table 1).

The univariate analysis revealed that TBIL levels were associated with the risk of complicated appendicitis (odds ratio: 1.098, 95% CI: 1.052–1.147). Serum sodium levels were also associated with the risk of complicated appendicitis (odds ratio: 0.743, 95% CI: 0.646–0.855). In contrast, sex was not associated with the risk of complicated appendicitis. These variables were included in a multivariate logistic analysis, which revealed that TBIL (odds ratio: 1.083, 95% CI: 1.033–1.135) and serum sodium (odds ratio: 0.789, 95% CI: 0.681–0.914) levels correlated with the risk of complicated appendicitis (Table 2). The Hosmer–Lemeshow goodness-of-fit *p*-value was 0.709.

**Figure 1:**
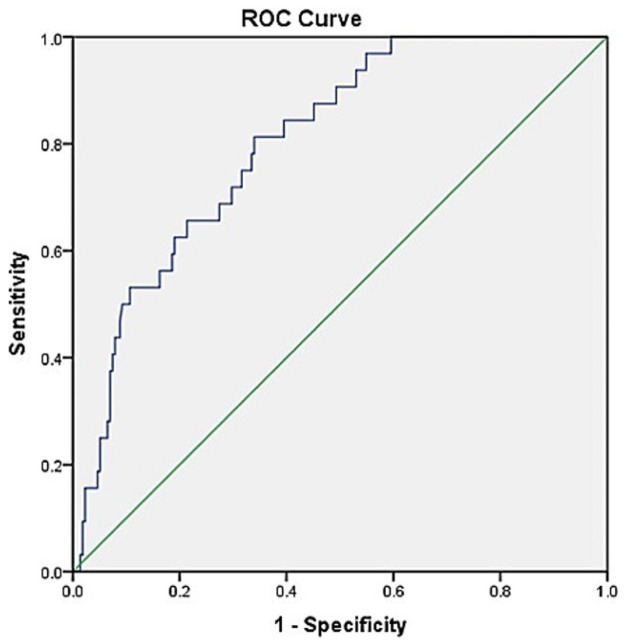
Receiver operating characteristic (ROC) curve to predict complicated appendicitis in a multivariate model.

To evaluate the diagnostic efficacy of hyperbilirubinemia, hyponatremia, and both combined, ROC analyses were conducted (Figure 1). Hyperbilirubinemia had sensitivity and specificity values of 65.6% and 75.4%, respectively. The positive predictive value (PPV) was only 28.4%, the negative predictive value (NPV) was 93.6%, and the AUC was 0.79. For hyponatremia, the sensitivity and specificity values were 84.4% and 45.6%, respectively. The PPV was 18.8%, the NPV was 95.1%, and the AUC was 0.73. Using hyperbilirubinemia in conjunction with hyponatremia improved diagnostic efficacy, yielding a sensitivity of 81.3%, a specificity of 64.7%, a PPV of 25.5%, an NPV of 95.9%, and an AUC of 0.80 (Table 3).

## 4. Discussion

Acute appendicitis is a common surgical emergency worldwide [22]. An early diagnosis of complicated appendicitis can significantly aid in ensuring a prompt surgical intervention, thereby improving clinical outcomes [7,23,24]. The advent of improved imaging methods, such as CT, has increased the diagnostic sensitivity for complicated appendicitis; however, the use of CT is expensive, cumbersome, and exposes patients to considerable radiation doses. Thus, identifying an accurate, easy to measure marker for the preoperative diagnosis of complicated appendicitis is of great importance. A number of studies have reported that hyperbilirubinemia (i.e., TBIL levels >20 µmol/L) has diagnostic potential for complicated appendicitis [11,13,14,20]. The present findings suggest that hyperbilirubinemia lacks a high enough sensitivity and specificity for the prediction of complicated appendicitis. Among the evaluated diagnostic variables for hyperbilirubinemia, only the NPV was above 90%. Recent studies have reported that hyponatremia (i.e., serum sodium levels of <135 mEq/L) is strongly associated with an increased risk of complicated appendicitis [16,17,18]. In the present study, hyponatremia exhibited high sensitivity for the prediction of complicated appendicitis, but it did not exhibit high specificity. Furthermore, the diagnostic efficacy of hyponatremia and hyperbilirubinemia improved when they were considered together.

Among the 247 patients with histologically confirmed appendicitis included in the present study, 13% had complicated appendicitis. The mean TBIL levels were 17.0 µmol/L in cases of early acute appendicitis and 23.3 µmol/L in cases of complicated appendicitis. Given that this study excluded patients with liver disease, liver transplantation, chronic alcoholism, congenital biliary disease, pregnancy, appendicular neoplasm, hemolytic disease, or known hepatocellular carcinoma, and that serum levels were measured preoperatively, the likely contributions of confounding factors to elevated TBIL levels were excluded. Moreover, we found that TBIL levels systematically increased with increasing appendicitis severity, suggesting a potential association between elevated TBIL levels and the severity of acute appendicitis [14]. Vaziri et al. also reported that the mean serum bilirubin level was higher in cases of acute perforated appendicitis than in cases of nonperforated uncomplicated acute appendicitis [25]. Hyperbilirubinemia may occur due to endotoxemia in both simple and perforated or gangrenous appendicitis [25]. Estrada et al. reported that the finding of elevated serum bilirubin levels in appendicitis depends on the pathogenesis of appendicitis [26]. Notably, mucosal ulceration occurs in early appendicitis and facilitates bacterial invasion into the muscularis propria of the appendix. Bacterial invasion activates the host immune system, leading to edema, inflammation, and increased intraluminal pressure. These changes lead to ischemic necrosis of the appendix, resulting in gangrene and favoring direct bacterial intrusion into the circulating bloodstream. Bacterial endotoxins interfere with the release of bilirubin into the bile ducts and subsequently lead to an elevation in serum bilirubin [26].

In the present study, increased TBIL levels were associated with an elevated risk of complicated appendicitis. However, this association exhibited only 65.5% sensitivity and 75.4% specificity. Furthermore, the PPV was only 28.4%, although the NPV was 93.6%. These findings do not support the use of TBIL as a discrete preoperative diagnostic marker. With respect to the diagnostic efficacy of hyperbilirubinemia, our findings differ considerably from those reported by Chaudhary et al. [14]. They reported 100% sensitivity and 100% PPV for TBIL levels of >1.2 mg/dL. In their study, all patients with gangrenous or perforated appendicitis had TBIL levels of >1.2 mg/dL. In contrast, in the present study, more than 50% of the patients with complicated appendicitis did not have hyperbilirubinemia. Given that the study conducted by Chaudhary et al. involved only eight patients with complicated appendicitis and that all of whom had hyperbilirubinemia, the observed high diagnostic efficacy was expected. Recently, Iftikhar et al. reported hyperbilirubinemia as a predictor for complicated appendicitis, with a sensitivity of 56.60% and a specificity of 95.45% [27]. Other recent studies also reported that elevated TBIL levels are associated with later stages of appendicitis [13,28]. A meta-analysis on the effect of elevated serum bilirubin on the likelihood of perforated appendicitis reported a sensitivity of 49% and a specificity of 82%, concluding that hyperbilirubinemia alone could not be used to distinguish perforated appendicitis, even though elevated TBIL levels had some predictive potential [11]. The present findings support this viewpoint: while elevated TBIL, in conjunction with other symptoms of complicated appendicitis, may be used to justify an appendectomy, hyperbilirubinemia alone does not seem to be a reliable indicator of complicated appendicitis.

Another potential diagnostic tool for complicated appendicitis is serum sodium [19,20]. Several recent studies reported an association between decreased serum sodium levels and an increased severity of appendicitis, increasing from early to complicated appendicitis [16,18,29]. In the current analysis, serum sodium levels varied substantially according to the severity of appendicitis. Hyponatremia as a predictor for complicated appendicitis exhibited 84.4% sensitivity and 45.6% specificity. In addition, the PPV was only 18.8%, although the NPV was 95.1%. These results indicate that the diagnostic efficacy of hyperbilirubinemia and hyponatremia improved when these markers were used in combination. Hyponatremia is an electrolyte disorder found in various diseases, such as pneumonia, tuberculosis, meningitis, encephalitis, human immunodeficiency virus, malaria, and dengue [30]. This condition can occur due to an early systemic inflammatory response mediated by interleukin-6 and vasopressin [17,30]. Heymowski et al. recently investigated the relationship between a plasma sodium threshold of 136 mEq/L and complicated appendicitis, and they found an odds ratio of 2.8 [31]. However, this value is considerably lower than the odds ratio of 3.2 reported in another recent study [32].

In terms of overall prevalence, the proportion of male patients was higher than the proportion of female patients, and this did not change with increased appendicitis severity. The patients had a mean age of 24.6 years and were more likely to be male than female. These findings are in line with those of prior studies [33,34]. Unlike Nshuti et al., we found no statistically significant difference in the prevalence of complicated appendicitis between male and female patients [35]. The majority of the patients in the present study had undergone a laparoscopic appendectomy, which is consistent with modern surgical practices [36,37].

The retrospective research design was the most apparent limitation of the present study. Given that we were only able to extract information available in the patients’ records, the possibility of errors cannot be completely ruled out, although the utmost care was taken in the data extraction process. The information we gathered was limited to the main variables related to the study objectives. It would be interesting to explore other clinical parameters and comorbidities in further studies. Notably, we excluded patients with liver dysfunction and related comorbidities. Finally, as this was a single-center study, the present findings need to be confirmed in large-scale prospective studies.

## 5. Conclusions

Hyperbilirubinemia and hyponatremia are important risk factors for complicated appendicitis. In the present study, the combination of hyperbilirubinemia and hyponatremia offered a discriminatory advantage. Our research contributes to the current body of knowledge by demonstrating that hyponatremia and hyperbilirubinemia, when combined, have a high NPV and are hence useful in ruling out complicated appendicitis. Conversely, the low specificity of these parameters shown by our study may caution doctors against relying too much on these parameters to confirm a diagnosis of complicated appendicitis. The results suggest that TBIL and serum sodium levels, along with other signs of complicated appendicitis, can be used as indications for an early appendectomy. These biomarkers may also be used to monitor treatment outcomes; however, large-scale investigations are necessary to verify our findings and explore their wider therapeutic implications.

## Figures and Tables

**Table 1:** Demographic and clinical characteristics according to the stages of appendicitis.

Variable	Early Acute (*n* = 36)	Acute Suppurative (*n* = 177)	Complicated (*n* = 32)	Other *(n* = 2)	*p*-Value
Age	23.9 ± 10.4	25.0 ± 12.4	23.2 ± 14.9	20.0 ± 5.7	0.801
	(15.0, 31.0)	(14.0, 33.0)	(14.5, 27.0)	(16.0, 24.0)	
Sex (male)	20 (55.6%)	116 (65.5%)	19 (59.4%)	1 (50.0%)	0.644
TBIL (µmol/L)	14.2 ± 5.1	17.0 ± 8.0	23.3 ± 6.3	6.8 ± 1.9	<0.001
	(11.0, 16.9)	(11.6, 20.9)	(17.7, 27.5)	(6.0, 8.7)	
Hyperbilirubinemia	5 (13.9%)	48 (27.1%)	15 (46.8%)	0 (0.0%)	<0.001
Serum sodium levels	135.9 ± 1.79	134.7 ± 2.7	132.6 ± 2.6	137.0 ± 2.8	<0.001
(mEq/L)	(135.0, 137.0)	(130.0, 134.0)	(130.0, 134.0)	(135.0, 139.0)	
Hyponatremia	13 (36.1%)	79 (44.6%)	22 (68.8%)	0 (0.0%)	<0.001
Hyperbilirubinemia + hyponatremia	1 (2.7%)	29 (16.4%)	18 (56.3%)	0 (0.0%)	<0.001
Appendectomy					0.743
Laparoscopic	34 (94.4%)	163 (92.1%)	31 (96.9%)	2 (100.0%)	
Open	2 (5.6%)	14 (7.9%)	1 (3.1%)	0 (0.0%)	

TBIL: total bilirubin; hyperbilirubinemia: TBIL > 20 µmol/L; hyponatremia: serum sodium level <135.0 mEq/L; continuous variables: mean ± SD (quartile 1, quartile 3) and Kruskal–Wallis test results; categorical variables: *N* (%) and Fisher’s exact test result.

**Table 2:** Logistic regression analysis for the risk of complicated appendicitis.

Variables	Univariate				Multivariate			
	OR	Lower	Upper	*p*-Value	OR	Lower	Upper	*p*-Value
Sex (male)	1.202	0.563	2.565	0.636	1.544	0.670	3.557	0.308
TBIL level (>20 µmol/L)	1.098	1.052	1.147	<0.001	1.083	1.033	1.135	<0.001
Serum sodium level (<135 mEg/L)	0.743	0.646	0.855	<0.001	0.789	0.681	0.914	0.002

**Table 3:** Diagnostic efficacy of the evaluated variables.

Variable	Sensitivity	Specificity	PPV	NPV	AUC
	(%, 95% CI)	(%, 95% CI)	(%, 95% CI)	(%, 95% CI)	(%, 95% CI)
Hyperbilirubinemia	65.6	75.4	28.4	93.6	0.79
	(46.8–81.4)	(69.0–81.0)	(22.0–35.8)	(90.1–96.0)	(0.737–0.842)
Hyponatremia	84.4	45.6	18.8	95.1	0.73
	(67.2–94.7)	(38.8–52.5)	(16.0–21.9)	(89.6–97.8)	(0.671–0.785)
Hyperbilirubinemia + hyponatremia	81.3	64.65	25.5	95.9	0.80
	(63.6–92.8)	(57.9–71.0)	(21.1–30.4)	(91.8–98.0)	(0.748–0.851)

## Data Availability

Not applicable.
